# A Case of Subacute Brain Hemorrhage and Disseminated Intravascular Coagulation Secondary to Acute Promyelocytic Leukemia in a Pediatric Patient

**DOI:** 10.7759/cureus.14922

**Published:** 2021-05-09

**Authors:** Mohamed E Elshazzly, Bilasan Hammo, Ilia N Buhtoiarov

**Affiliations:** 1 Pediatrics, Cleveland Clinic Foundation, Cleveland, USA; 2 Pediatric Hematology/Oncology, Cleveland Clinic Foundation, Cleveland, USA

**Keywords:** acute subdural hematoma, disseminated intravascular coagulation (dic), acute promyelocytic leukemia, apml, pediatric

## Abstract

Acute promyelocytic leukemia (APML), characterized by the reciprocal translocation between chromosomes 15 and 17 [t(15;17)], is a result of proliferation of myeloid cells maturation which is interrupted at the promyelocytic stage. The central, and the most important, distinguishing feature of APML is a predisposition to disseminated intravascular coagulation (DIC). The overall prognosis of APML is very good, with 90% of patients achieving complete remission. We find it important to remind pediatric practitioners, both in the ambulatory and urgent care room settings, of presenting signs and symptoms of leukemia, as well as, up-to-date on management of such fulminant scenarios as DIC. Intracranial hemorrhage (ICH) is one of the commonest, and frequently fulminant complication of APML seen after initiation of induction chemotherapy. We report on a young female presenting with non-specific upper respiratory illness symptoms and recurrent headache, who was found to already have ICH and to be in DIC in the setting of APML at the time of initial evaluation. This case illustrates importance of thorough assessment and prompt consideration of wide differential diagnosis, which became somewhat limited and biased towards web-based telemedicine in the COVID-19 pandemics era.

## Introduction

In the United States, it has been reported that 600-800 new cases of acute promyelocytic leukemia (APML) are diagnosed each year, with the majority of cases reported in adults [1]. It is noted that APML mostly affects the pediatric population between nine and 12 years old [2]. Children with APML commonly present with symptoms related to corresponding cytopenias, which are also commonly seen in other types of acute leukemia. Children, in particular, who are afflicted with APML often present with vague and non-specific symptoms, posing a challenge to early diagnosis and management. One of the key hallmarks and the distinguishing feature of APML is profound coagulopathy, manifesting as disseminated intravascular coagulation (DIC). Given that a patient can deteriorate very acutely, misdiagnosing or even completely missing the diagnosis of DIC in an affected patient can cause significant morbidity and mortality. If recognized and treated emergently, patients with APL have excellent survival outcome.

## Case presentation

A previously healthy, fully vaccinated, 15-year-old female presented to the emergency department (ED) for evaluation of 24-hour long history of fever, emesis and diarrhea. One week prior to this visit, the patient was seen by primary care physician for headache, cough and nasal congestion, and was prescribed a therapy with amoxicillin/clavulanate for presumed sinusitis. In the ED, the patient endorsed fatigue, recurrent headaches, and multiple ecchymoses on lower extremities that appeared without any inciting trauma. Furthermore, the patient had 12-pound weight loss over the past three months, which was attributed to family and school stressors. Previous history of abdominal or musculoskeletal pain, or excessive mucocutaneous bleeding was denied. The family history was otherwise significant for depression and migraine headaches in patient’s mother but negative for bleeding diathesis. Patient’s father endorsed recent exposure to SARS-CoV-2; however, he did not experience any fever, respiratory, gastrointestinal or mucocutaneous eruptions.

At presentation, the patient was tired-appearing; febrile to 102.4F/39.1C, tachycardic to 110 beats/min, and mildly tachypneic to 23 respirations/min, but normotensive for age. Physical examination was unremarkable except for parched lips, tongue, and oral mucosa, and multiple ecchymoses in different stages of involution. No focal neurological deficits were documented.

Initial laboratory blood tests findings revealed severe anemia with hemoglobin level of 5.5 g/dl (55 g/L) [range: 12-15.5 g/dl], thrombocytopenia with platelet count of 13 x 10^3^/µl (13 x 10^9^/L) [range: 140-400/µl], and white blood cell count of 7900/µl (7.9 x 10^9^/L) [range: 4.5-11.0 x 10^9^/L]. Coagulation panel studies were significant for prolonged prothrombin time (PT) of 17.2 sec; prolonged international normalized ratio (INR) of 1.6; fibrinogen of 62 mg/dL (0.62 g/L), but with a normal partial thromboplastin time (PTT) of 26 sec. D-dimer was 10,880 ng/mL (normal values <500 ng/mL).

**Figure 1:**
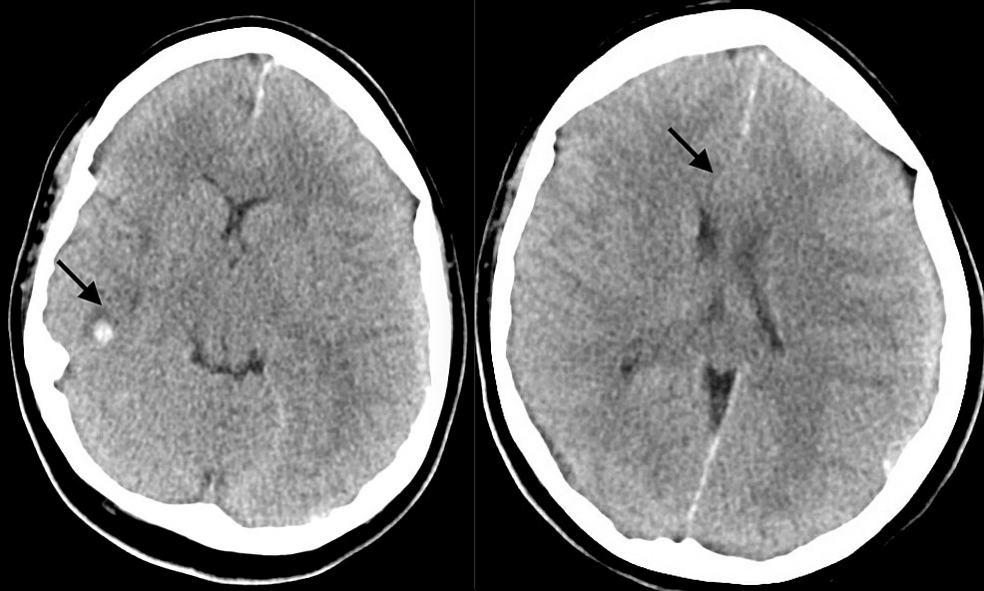
CT images with overall findings concerning for acute on subacute left hemispheric subdural hematoma Image on left: Arrow pointing to 6 mm hyperdense focus right temporal lobe inferiorly with adjacent vasogenic edema concerning for small parenchymal hematoma. Image on right: There is an extra-axial collection over the left frontoparietal region with maximal thickness of 6 mm and mass effect resulting in 4 mm left-to-right midline shift.

**Figure 2:**
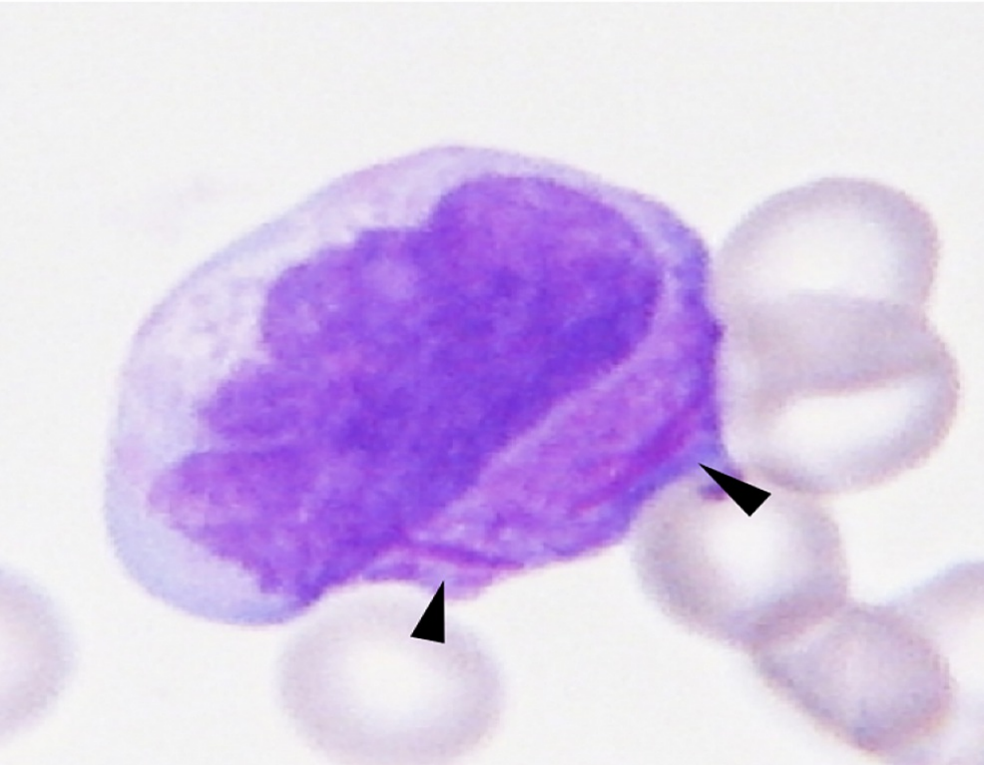
Peripheral blood smear revealed presence of atypical hypergranular cells with large needle-shaped inclusion bodies in the cytoplasm, consistent with Auer rods, typically seen in APL. They are strongly positive for myeloperoxidase.

A non-contrast computed tomography (CT) of the head, which was obtained to address the etiology of recurrent headaches demonstrated left acute on subacute subdural hematoma with mass effect resulting in 4 mm left-to-right midline shift and a 6-mm right temporal lobe hematoma (Figure 1). Staff review of peripheral blood smear revealed presence of atypical hypergranular cells with large cytoplasmic inclusion bodies, consistent with Auer rods (Figure 2). The patient was emergently transferred to the pediatric intensive care unit (PICU) for hyperacute management of DIC. Detailed analysis of lymphocyte subpopulations by flow cytometry revealed 30% blasts and 10% promyeloblasts, consistent with acute promyelocytic leukemia (APML) pathomorphological diagnosis.

**Figure 3:**
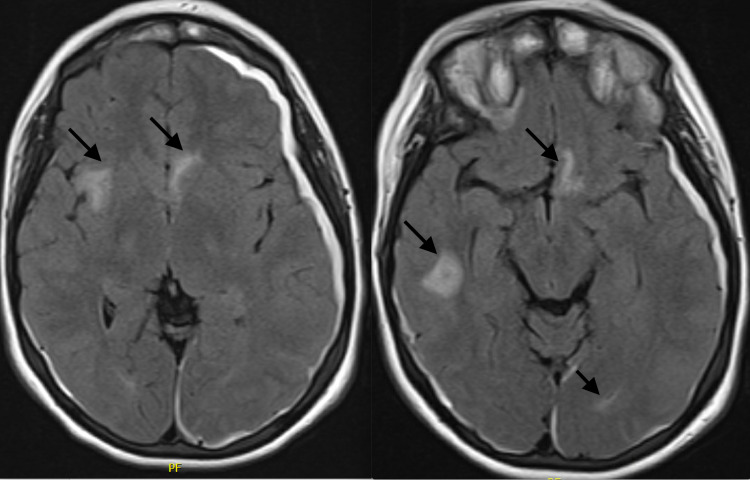
MRI findings: Similar findings to CT scan. Arrows in both images pointing to multifocal areas of intracranial hemorrhage

While in the PICU, the patient did not exhibit any worsening of neurological or circulatory functions. Brain MRI confirmed presence of subdural bleeding but also revealed additional parenchymal microhemorrhages (Figure 3). No emergency neurosurgical intervention was warranted due to absence of neurological signs and symptoms, as well as lack of evidence of radiographic progression on subsequent brain imaging. The patient urgently received infusion of packed red blood cells, platelets, and cryoprecipitate; first dose of all-trans-retinoic acid (ATRA) was also rapidly administered.

## Discussion

In the ED setting, a patient presenting with fever, emesis, diarrhea, and signs of dehydration but otherwise well-appearing, could have been remediated for viral gastroenteritis. Given this patient’s family history of migraine headaches, and the patient’s presentation with headache and emesis, a migraine headache was also on the differential. Complicated rhinosinusitis was considered but was less likely given improvement in the symptoms (cough and nasal congestion), and the patient almost completing the 10-day course of amoxicillin/clavulanate. Also, amoxicillin/clavulanate is known to cause gastrointestinal distress as one of its side effects, and isolated thrombocytopenia, although unlikely the cause given the rest of this patient’s unexplained symptoms.

While the viral gastroenteritis and migraine headaches were on top of working differential diagnosis, the presentation of a teenager with recurrent headache, recurrent fevers, and ecchymoses warranted a detailed investigation. Hence, complete blood count (CBC) indexes and coagulation studies were obtained and demonstrated severe anemia, thrombocytopenia, prolonged PT/INR, low fibrinogen, and high D-dimer consistent with DIC. Due to the chronic headache and severe thrombocytopenia, the patient received a CT scan of the head, which showed an acute on chronic intracranial bleeding. Given the bicytopenia and intracranial hemorrhage (ICH) on emergency imaging, the patient’s presentation was concerning for a hematological process such as acute leukemia, viral-induced bone marrow suppression, or idiopathic bone marrow failure. With a constellation of the headaches, fatigue, fever, weight loss, and intracranial bleed, in the setting of bicytopenia and evolving DIC, the patient’s critical presentation was concerning for APML.

APML, also known as the M3 subtype of acute myelogenous leukemia (AML) in the French-American-British (FAB) classification, is a result of clonal proliferation of myeloid cells interrupted at the promyelocytic stage [1]. This occurs due to the characteristic translocation of the promyelocytic leukemia (PML) gene on chromosome 15 and the Retinoic Acid Receptor-alpha (RARA) gene on chromosome 17, [t(15;17)(q22;q21.1)]” [1-2]. On peripheral blood smear, APML blast cells have a pathognomonic appearance showing incased cytoplasmic granules, bi-lobed nuclei, and defined Auer rods, which are fused lysosomes rich of various lysosomal enzymes forming needle-like shapes. Worsening fatigue, recurrent fevers, pallor, and mucocutaneous bleeding are frequent complaints [3]. The overall prognosis of APML is extremely good, with a large percentage of patients (greater than 90%) achieving complete remission and five-year overall survival rates over 80% [4-5].

**Figure 4:**
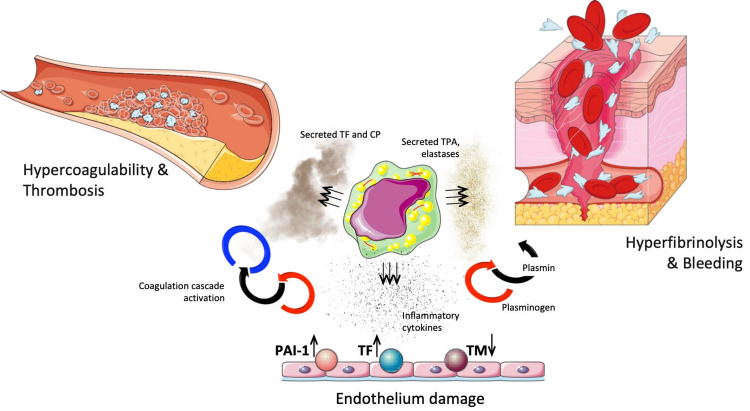
The pathogenesis of disseminated intravascular coagulation (DIC) in acute promyelocytic leukemia (APML) is very complex. APML cells produce inflammatory cytokines, such as IL-1β, IL-6, TNF-α, which damage endothelial cells and change expression of tissue factor (TF), thrombomodulin (TM), and plasminogen activator inhibitor-1 (PAI-1), thereby altering blood homeostasis and triggering coagulation cascade. Furthermore, APML blasts themselves express TF and various cysteine proteinases, also known as cancer procoagulants (CP), which lead to independent activation of the coagulation cascade. On the other end, APML blasts produce tissue plasminogen activator (TPA) and various elastases, which trigger fibrinogen degradation resulting in hyperfibrinolysis and uncontrolled bleeding.

The central, and the most important, distinguishing feature of APML is a predisposition to DIC. The pathogenesis of DIC is quite complex, and involves states of self-propagating hypercoagulation, hyperfibrinolysis and microvascular endothelial damage (Figure 4). The vast majority of APML patients will initially present with a “bleeding diathesis” [6-8]. Classically, DIC is defined by prolonged PT/INR and PTT, low fibrinogen, elevated D-dimer and other fibrin degradation products, and thrombocytopenia (Figure 4). Consequently, DIC is well known to be associated with APML [9-10]. Intracranial hemorrhage (ICH) is the most critical and most frequent complication of APML, acutely affecting the survival of APML patients during early course of disease and therapy [11-12].

ATRA has revolutionized the treatment of APML; it induces rapid differentiation (i.e., maturation) of leukemic promyeloblasts into mature granulocytes leading to a quick correction in coagulopathies [13]. Starting ATRA therapy as soon as APML is clinically suspected is crucial to reduce complications of ICH such as death; most fatal ICH occurs within 10 days of diagnosis [14]. One of the remarkably unusual aspect of this case is that ICH was a presenting sign before the patient received any leukemia-specific treatment, and not a complication of APML treatment.

**Figure 5:**
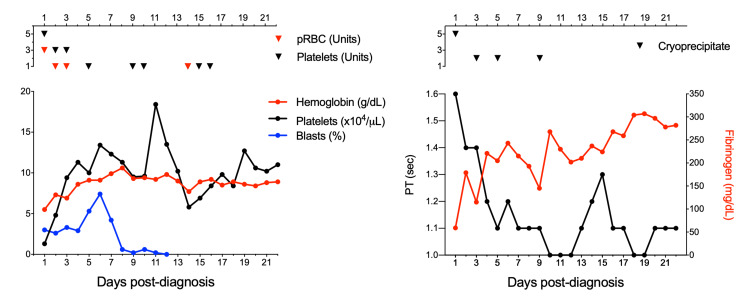
Blood monitoring during 23 days of hospitalization Left pane: X-axis shows days post-diagnosis. Y-axis shows laboratory values. Red line indicates hemoglobin levels. Black line indicates platelet levels. Blue line indicates Blast percentages. pRBC: packed red blood cells. Right pane: X-axis shows days post-diagnosis. Black line and left Y-axis show PT values (time in seconds). Red line and right Y-axis show fibrinogen levels.

In conjunction with emergent ATRA therapy, DIC should be treated aggressively and immediately. Fresh frozen plasma and cryoprecipitate should be transfused to maintain INR below 1.5 and fibrinogen levels greater than 150 g/dL, respectively; platelets should be transfused to maintain platelet counts over 50 x 10^9^/L [15]. On average, it takes a median time of four days for the coagulopathy in APML to correct after starting treatment with ATRA [4]. Our patient required aggressive transfusion support for a total of 16 days (Figure 5). Providers must also need to be aware that ATRA has notable side effects including pseudotumor cerebri and differentiation syndrome (formerly known as retinoic acid syndrome). Furthermore, initiation of ATRA may exacerbate DIC initially, and can increase risk of ICH. Close attention during induction is important, and symptoms of headaches should have a low threshold for suspicion of ICH.

The current standard of care regarding children with APML is induction with both ATRA and arsenic trioxide (ATO), followed with four cycles of consolidation therapy [16]. This ATRA+ATO combination allows achieving complete response and durable complete remission in 96% of cases [17-18]. Classical chemotherapy agents with anti-leukemia potential such as idarubicin, prednisone and hydroxyurea are used for treatment of high-risk conditions [18]. Recent advances in understanding biology of APML led to development of additional treatment approaches, such as Tamibarotene (a synthetic retinoid with mechanism of action similar to ATRA), and gemtuzumab ozogamicin, an antibody-drug conjugate of anti-CD33 monoclonal antibody linked to cytotoxic agent from the class of calicheamicins [19]. Stem cell transplantation is reserved for the standard treatment-refractory cases.

## Conclusions

Our patient’s acute presentation highlights the importance of early diagnosis of APML. Given the preceding signs and symptoms of upper respiratory infection or sinusitis and presumed exposure to SARS-CoV-2, this could have been easily overlooked. A patient presenting with recurrent headache, weight loss, mucocutaneous hemorrhagic eruptions, and clinico-laboratory findings concerning for DIC is APML unless proven otherwise.

Even though our patient had very vague and non-specific symptoms, it is imperative to build an appropriate differential based on ascertaining acute from chronic symptoms. This can prompt, then, earlier imaging and focused hematological testing. Hence, by this case report we intend to sharpen diagnostic intuition of medical providers, in the era of encouragement of limited contacts and web-based telemedicine for common illness management.
